# Characterization of heterotypic interaction effects *in vitro *to deconvolute global gene expression profiles in cancer

**DOI:** 10.1186/gb-2007-8-9-r191

**Published:** 2007-09-14

**Authors:** Martin Buess, Dimitry SA Nuyten, Trevor Hastie, Torsten Nielsen, Robert Pesich, Patrick O Brown

**Affiliations:** 1 Department of Biochemistry, Stanford University School of Medicine, Stanford, CA 94305, USA; 2 Department of Statistics, Stanford University School of Medicine, Stanford, CA 94305, USA; 3 Howard Hughes Medical Institute, Stanford University School of Medicine, Stanford, CA 94305, USA; 4 Departments of Radiation Oncology and Diagnostic Oncology, Netherlands Cancer Institute, 1066 CX Amsterdam, The Netherlands; 5 Department of Pathology and Laboratory Medicine, University of British Columbia, Vancouver, BC, Canada, V5Z 1M9

## Abstract

**Background:**

Perturbations in cell-cell interactions are a key feature of cancer. However, little is known about the systematic effects of cell-cell interaction on global gene expression in cancer.

**Results:**

We used an *ex vivo *model to simulate tumor-stroma interaction by systematically co-cultivating breast cancer cells with stromal fibroblasts and determined associated gene expression changes with cDNA microarrays. In the complex picture of epithelial-mesenchymal interaction effects, a prominent characteristic was an induction of interferon-response genes (IRGs) in a subset of cancer cells. In close proximity to these cancer cells, the fibroblasts secreted type I interferons, which, in turn, induced expression of the IRGs in the tumor cells. Paralleling this model, immunohistochemical analysis of human breast cancer tissues showed that STAT1, the key transcriptional activator of the IRGs, and itself an IRG, was expressed in a subset of the cancers, with a striking pattern of elevated expression in the cancer cells in close proximity to the stroma. *In vivo*, expression of the IRGs was remarkably coherent, providing a basis for segregation of 295 early-stage breast cancers into two groups. Tumors with high compared to low expression levels of IRGs were associated with significantly shorter overall survival; 59% versus 80% at 10 years (log-rank *p *= 0.001).

**Conclusion:**

In an effort to deconvolute global gene expression profiles of breast cancer by systematic characterization of heterotypic interaction effects *in vitro*, we found that an interaction between some breast cancer cells and stromal fibroblasts can induce an interferon-response, and that this response may be associated with a greater propensity for tumor progression.

## Abstract 2

In an effort to deconvolute global gene-expression profiles, an interaction between some breast cancer cells and stromal fibroblasts was found to induce an interferon response, which may be associated with a greater propensity for tumor progression.

## Background

Communication between different cell types is fundamental for the development and homeostasis of multi-cellular organisms. Cells of different origin communicate in a network of interactions via proteins, peptides, small molecular signals, the extracellular matrix and direct cell-cell contact. These heterotypic interactions provide information that is necessary for the regulation of the gene expression programs in normal development [1], differentiation [2], topologic organization [3] and homeostasis [4] of complex tissue structures. Given the important physiological role of intercellular communication to maintain the delicate dynamic equilibrium of a normal tissue, it is not surprising that aberrant cell-cell interaction signals have been implicated in cancer development and progression [5-10]. Although the characteristics and roots of the heterotypic interaction effects are fundamental aspects of normal physiology and disease, they have not been systematically explored.

In cancer biology, there is increasing evidence for the importance of the interaction between the malignant epithelial cells and the surrounding stromal cells [7]. Tumors are not merely aggregates of malignant cells but are in many respects organ-like structures, which include host stromal cells, such as fibroblasts, endothelial cells and so on, with which the malignant cells themselves intermingle and interact. Inductive interactions between these different cell lineages can play not only a morphogenetic role but also an important mechanistic role in the pathogenesis and progression of malignancy. Co-inoculation of stromal cells with pre-malignant or malignant epithelial cells can increase tumorigenicity and the capacity to metastasize for a variety of tumor types [11,12], including breast cancer [13]. On the molecular level, results from the knockout of single genes have demonstrated the importance of specific signaling pathways in the tumor-stroma interaction. For example, conditional inactivation of the transforming growth factor (TGF)-β receptor type II in stromal cells led to development of epithelial cancer of the prostate and forestomach in mice [14]. In the mammary gland, site-specific knockout of TGF-β receptor type II in stromal fibroblasts led to defective mammary ductal development and increased carcinoma growth and metastasis [15]. Experiments exploring the interaction of tumor with stromal cells *in vitro *have revealed changes in expression of several genes involved in cancer [16-18]. These effects reveal the significance of one specific signaling mechanism, but a more complete overview of the molecular systems that mediate these cell-cell interaction effects remains to be revealed.

Biopsy samples of human carcinoma frequently contain both malignant cells and stromal cells. Since gene expression profiles of human cancer are generally derived from these mixed cell populations of grossly dissected tissues, the effects of heterotypic interactions among the cells in the tumor tissue are expected to leave their traces in the global gene expression profiles. Datasets representing expression profiles of thousands of genes in collections of benign and malignant tissues from hundreds of patients have steadily grown in recent years and might be a rich latent source of insights into heterotypic interaction effects on global gene expression. The superposition of the cell specific profiles, however, results in complex gene expression patterns that are difficult to interpret. In breast cancer, Allinen *et al*. [19] attempted to resolve this complexity by fractionating the tissue using cell-surface markers to separate different cell types. This led to the identification of cell type specific gene expression profiles. As a result of this analysis they suggested that a myofibroblast expression of CXCL14 and CXCL12, which can bind to the respective receptor CXCR4 on the epithelial cells, is a specific tumor promoting mechanism leading to enhanced proliferation, invasion and metastasis. In a different approach to search for the relevance of stromal signals in cancer data, West *et al*. [20] identified stromal-cell specific gene expression signatures in breast cancer using gene expression data from fibroblastic tumors as *in vivo *models of homogenous populations of malignant mesenchymal cells. Based on stromal-cell specific signatures they were able to segregate breast cancer samples into two subgroups with distinct clinical outcome.

A further layer of complexity, in addition to the simple additive effects of the involved cells, might arise from the effects on gene expression profiles induced by heterotypic cell-cell interactions. The deconvolution of these intercellular signaling effects poses an even greater challenge, since they result in supra-additive non-linear behaviors, which are hard to disentangle and distinguish from the cell-intrinsic regulatory processes. These cell interaction effects might account for a significant proportion of the unrevealed information in the gene expression data from tissue specimens. Given the evidence that interactions between cells can play critical roles in tumor progression, such data might be even more meaningful than prominent expression patterns that are driven by the proportional representation of a given cell type in a tissue [21].

The primary aim of this work was to survey and characterize the effects of cell-cell interaction in an attempt to disentangle the complex network of intercellular signaling in a multi-cellular tissue and specifically in breast cancer. To extract the information about tumor-stroma interaction from global gene expression profiles of cancer tissue, we applied an approach based on *in vitro *modeling combined with subsequent testing of the *in vitro *findings in published cancer datasets. Observation of fundamental biological processes *in vitro*, such as the cell cycle [22] and the reaction of fibroblasts to serum [23,24], or observation of the common response of different cell types to hypoxic conditions [25] has proven to be a worthwhile approach to better understand complex biological mechanisms underlying global gene expression profiles in human cancer. Using a simple *ex vivo *co-culture system allowed us to address a few basic questions about heterotypic cell-cell interactions. First, is global gene expression in a co-culture setting different from the expression in monoculture and, if so, in which respect is it different? Second, how do the responses to co-culture differ among different cell combinations? Third, are the *in vitro *observations transferable *in vivo *using published gene expression datasets from human tissue specimens? We analyzed heterotypic interaction effects in stromal fibroblasts and a diverse set of benign and malignant breast epithelial cells in a mixed co-culture setting by measuring changes in global gene expression using DNA microarrays. The global view of the gene expression responses facilitated the identification of specific changes and pathways underlying these effects. Gene expression signatures paralleling a response to heterotypic interaction in this *ex vivo *model were shared by clinically distinct subgroups of breast cancer.

## Results

### Identification of heterotypic interaction effects

**Figure 1:**
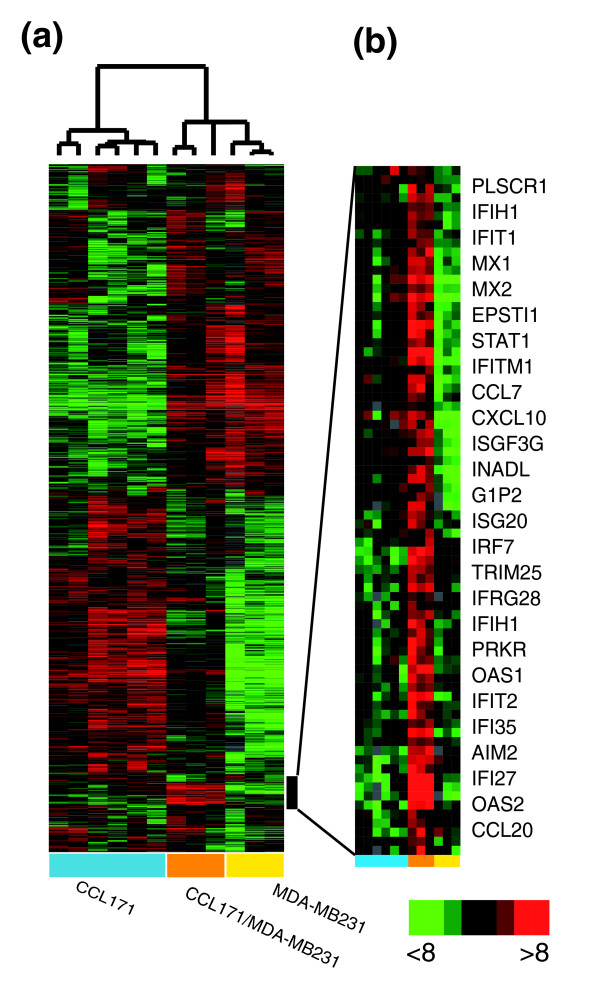
Effect of heterotypic interaction between breast cancer cell line MDA-MB231 and CCL-171 fibroblasts. **(a) **Biologically independent replicates of the monocultured fibroblast CCL-171, the breast cancer cell line MDA-MB231 and the mixed co-culture of CCL-171 and MDA-MB231 were grown for 48 h at low serum conditions and characterized by DNA microarray hybridization. Hierarchical clustering of a total of 4,333 elements that display a greater than 3-fold variance in expression in more than 3 different experimental samples. Data from individual elements or genes are represented as single rows, and different experiments are shown as columns. Red and green denote expression levels of the samples. The intensity of the color reflects the magnitude of the deviation from baseline. Unsupervised hierarchical clustering of the experiments grouped the biological replicates together. Gene expression varied considerably between fibroblast and MDA-MB231 cultures, as expected for cells of mesenchymal or epithelial origin, respectively. The co-culture profile showed mainly intermediate expression levels. However, the vertical black bar marks a cluster of genes induced in all co-cultures compared to both monocultures, indicating that they are induced by heterotypic interaction. **(b) **Zooming in on the genes up-regulated in co-culture compared to monocultures reveals that they are associated with the response to interferon.

As a model for investigating the gene expression program in response to heterotypic cell-cell interaction in normal breast and in breast cancer, we examined cells representing the benign and malignant epithelial cell compartment and the mesenchymal cell compartment in an *in vitro *mixed co-culture setting. The cells were co-cultivated for 48 h in low fetal bovine serum medium (0.2% FBS) to allow reciprocal signal exchange with minimal background from the influence of undefined molecular signals inherent in FBS. We examined the effects of co-cultivation for each cell pair in at least two independent biological replicates. The gene expression profiles of the co-cultures were compared to the expression profiles of the corresponding cells kept in monoculture using cDNA microarrays containing approximately 40,700 elements, representing 24,472 unique Unigene clusters (build number 173, released on 28 July 2004). To establish the experimental approach, we first focused our experiments on the breast cancer cell line MDA-MB231, the primary fibroblast CCL-171 and the co-culture of these two cell types. The data were organized using unsupervised hierarchical clustering of the replicate experiments to provide an overview of the effects on global gene expression (Figure 1a). In the co-culture, most genes displayed intermediate expression levels, which closely approximated the proportionally weighted average of their expression levels in the two cell types in monoculture. However, one set of genes showed a consistent, significant increase in transcript abundance in the co-culture compared to either monoculture, suggesting that induction of these genes was an effect of co-cultivation. Most of these induced genes were known to be interferon regulated (Figure 1b). They included those encoding the myxovirus resistance proteins 1 and 2 (MX1 and MX2), 2',5'-oligoadenylate synthetase 1 and 2 and 3 (OAS1, OAS2, OAS3) and interferon-induced protein with tetratricopeptide repeats 1 (IFIT1), phospholipid scramblase 1 (PLSCR1), eukaryotic translation initiation factor 2-alpha kinase (EIF2AK2) and the signal transducer and activator of transcription (STAT1). One of these genes, *EPSTI1*, had previously been reported to be induced by co-cultivation of MDA-MB231 and a fibroblast [17]. Our results suggest that the interferon response pathway mediates this induction. Although several of the genes induced in this co-culture model have not previously been linked to interferon induction (for example, *zinc finger protein 187 (ZNF187), Homo sapiens peroxisomal proliferator-activated receptor A interacting complex 285 (PRIC285), hect domain and RLD 6 (HERC6)*), we have confirmed that they are induced in MDA-MB231 cells by treatment with a type I interferon. As a more explicit approach to identify genes with consistent changes in expression in response to co-culture we used significance analysis of microarrays (SAM) [26]. A set of 42 genes represented by 49 image clones were identified with a false discovery rate (FDR) of 0 (Additional data file 1).

To further validate the results obtained by cDNA microarray analysis *OAS2 *transcript levels were measured by quantitative real time PCR (Figure A in Additional data file 2). Moreover, for *STAT1 *the increase in transcript levels in co-culture (2.8-fold) was paralleled by an increase in STAT1 protein as detected by fluorescence assisted cell sorting (FACS) analysis (Figure B in Additional data file 2).

Since breast cancer is a clinically and molecularly heterogeneous disease, we selected a broad spectrum of different breast cancer cell lines to sample this heterogeneity and explored the effects of heterotypic culture looking for subtype-specific and shared response patterns. We focused on epithelial-mesenchymal interactions co-cultivating fibroblasts of different origins (HTB125 (breast stromal fibroblast), HDF (fibroblast from breast skin) and CCL-171 (embryonic lung fibroblast)), in combination with normal breast epithelial cells (human mammary epithelial cells (HMECs)) and seven widely used breast cancer cell lines.

**Figure 2:**
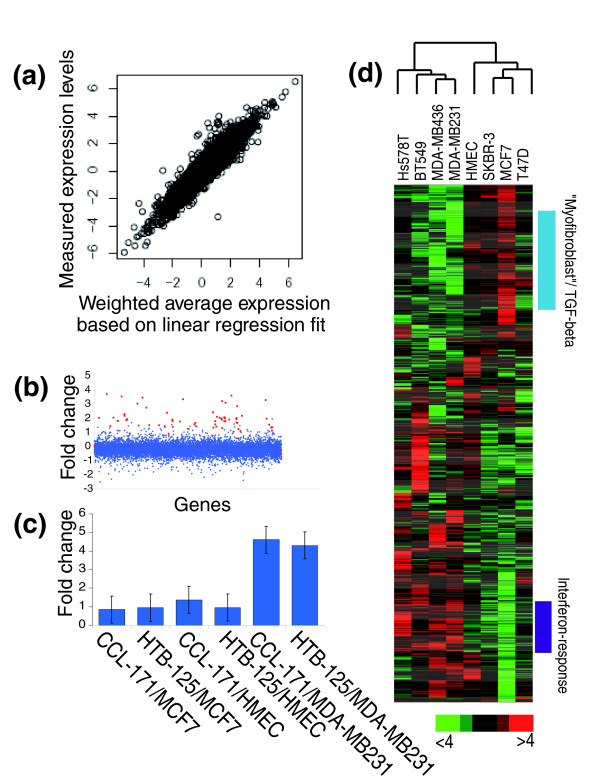
Overview of gene expression changes over multiple co-cultures of breast cancer cell lines and normal breast epithelial cells with fibroblasts. **(a) **Correlation of the measured co-culture gene expression levels and their estimated expression levels based on the proportional contribution of each cell type determined by a linear regression fit of the monoculture to the co-culture data. **(b) **Fold change of each gene associated with co-culturing of MDA-MB231 and CCL-171. Genes of the interferon response gene set (Additional data file 1) as determined by SAM are indicated in red. **(c) **Fold change in expression of the interferon response gene set (Additional data file 1) in co-culture of MCF-7, HMECs and MDA-MB-231 with either the CCL-171 lung fibroblast or the HTB-125 breast fibroblast, showing that CCL-171 and HTB-125 induce a distinct, but very similar response in co-culture with different epithelial cells. **(d) **Overview of collapsed data from repeat co-culture experiments of eight benign and malignant epithelial cells with three different fibroblasts. Hierarchical clustering of the interaction effects of 3,000 genes in co-cultures of 7 breast cancer cell lines and normal breast epithelial cells with fibroblasts. Red and green denote relative changes in expression associated heterotypic interaction. The magnitude of the relative change is given by color intensity.

The changes in gene expression due to heterotypic interaction were subtle compared to the large intrinsic variation in expression patterns among the involved cell types, as Figure 1a illustrates for the cell pair MDA-MB231 and CCL-171. To identify the gene expression changes resulting from cell-cell interaction, we needed to control for the simple additive effects that reflect the proportional contribution of the two cell types to the total population of each gene's transcript in the co-culture. Eliminating these proportionally weighted additive contributions would allow us to isolate supra-additive interaction effects. The fact that transcript levels of most genes did not change in response to co-culture allowed a linear regression model based on the transcript profiles of each monoculture to be fitted to the co-culture data for normalization. An example of such an analysis is shown in Figure 2a. For each gene, the ratio of the measured transcript level and the level estimated by the linear model provides a measure of the heterotypic interaction effect. This is illustrated in Figure 2b, which shows the distribution of the gene expression changes of the CCL-171/MDA-MB231 co-culture. The genes identified by SAM as differentially expressed in co-culture compared to monoculture are highlighted to illustrate the performance of this approach. Interaction effects, represented as gene-expression changes, are converted to quantitative values that can be analyzed for similarities and disparities over multiple different pair-wise interactions between cells with the same tools we use to analyze conventional gene expression data.

There was obvious heterogeneity in the responses of different pairs of cells to co-cultivation. The patterns of gene expression changes due to co-cultivation were mainly determined by the type of the epithelial cell involved whereas the origin of the fibroblasts had a minor influence. Importantly, CCL-171, a lung fibroblast, and HTB125, a fibroblast derived from the breast of a cancer patient, induced distinct but very similar interferon responses in co-cultivation with different epithelial cells (Figure 2c). To highlight consistent features of the responses of distinct normal or malignant epithelial cells, representing the distinct types of breast cancer, to co-cultivation with fibroblasts, we collapsed our data into eight groups, one group for each epithelial cell co-cultured with three different types of fibroblasts. There were 3,000 genes that showed a significant reproducible change (FDR < 1%) in transcript levels in response to co-culture in at least one of the groups. Clustering the averaged values of co-culture-induced changes for each group revealed specific and shared effects (Figure 2d). For several cell combinations, co-cultures led to an induction of *smooth muscle actin (ACTA2), myosin regulatory light chain interacting protein (MYLIP), myosin, light polypeptide kinase (MYL), myosin regulatory light chain 2, smooth muscle isoform (MYL9), calponin 2 (CNN2) *and *fibronectin (FN1)*. Induction of these genes has previously been described to be associated with the acquisition of a myofibroblast phenotype [27]. The ability of the tumor cells to induce this 'myofibroblast' expression program varied among the breast cancer cell lines; the strongest effect was seen with MCF7 cells. In a previous study, conditioned medium of MCF7 cells was shown to induce a myofibroblast phenotype [28]. Targets of the TGF-β pathway, such as the gene encoding *latent transforming growth factor beta binding protein LTBP2 *and transforming growth factor induced gene TGFBI, were induced in parallel with the 'myofibroblast response'. In fact, TGF-β has previously been shown to induce a 'myofibroblast' phenotype [29], suggesting that the response observed in these co-cultures might be mediated by the TGF-β pathway.

The most consistent coordinated response, however, was an induction of interferon-associated genes by cultivation of fibroblasts with four of the seven breast cancer cell lines. This response was seen in the co-cultures involving the estrogen-receptor negative breast cancer cell lines MDA-MB231, MDA-MB436, Hs578T and BT549, but neither in HMECs nor in the estrogen-receptor positive breast cancer cells MCF7, T47D and SKBR-3. Although the gene expression profiles of these epithelial cells grown as monocultures reflected their molecular differences, including some consistent differences between the estrogen-receptor negative and estrogen-receptor positive breast cancer cell lines, there were no consistent differences between these groups in baseline expression of the interferon-induced genes in the monocultures. The cell-type specificity is a strong hint that the interferon-response activation is a specific effect of heterotypic interaction. Since we compared the gene-expression responses in the co-cultures with the responses in the corresponding monocultures kept under the same conditions, we can exclude responses to serum stimulation or withdrawal as sources of the interferon response observed in these experiments. The response does not represent an effect of crowding, which is a known inducer of an interferon response [30], since the cell density in our experiments was maintained below the threshold at which the interferon response genes were turned on (data not shown)**. **Furthermore, we were unable to identify any infective agent in any of the cultures despite extensive testing for mycoplasma, reverse transcriptase activity and viral transcripts, using microarrays that provide a broad survey of human viruses [31] (data not shown). The consistent cell-type specific, coordinated response suggests that it depends on a specific physiological feature shared among the estrogen-receptor negative human breast cancers, which is retained in long-term culture, enabling them to activate this specific response upon contact with stromal cells.

### Localizing expression of interferon-response genes to breast cancer cell lines

**Figure 3:**
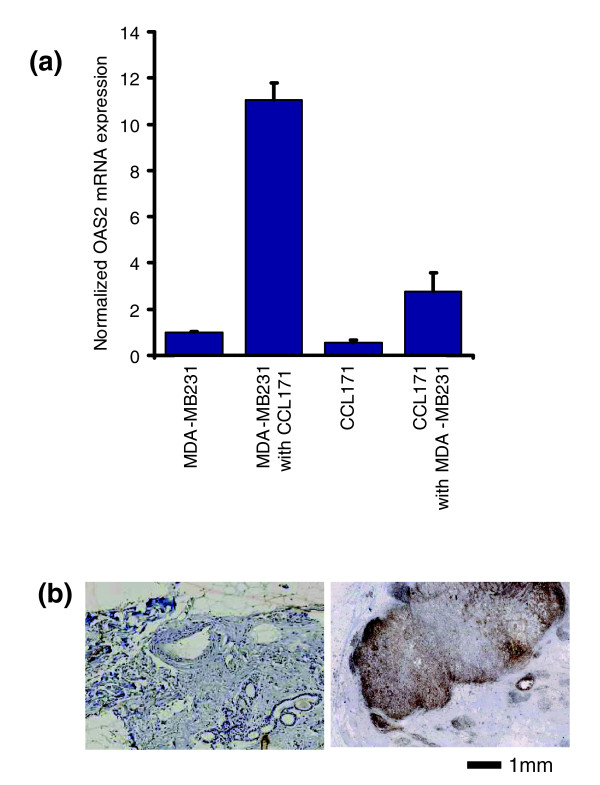
Interferon response gene induction in co-cultivated cells. **(a) **MDA-MB231 breast cancer cells and CCL-171 fibroblasts were labeled before co-culture with the fluorescent carbocyanine dye DiO and isolated after co-culture using FACS, which allowed a purification of 95%. Comparing gene expression patterns of the cells cultivated in monoculture to the same cell type after co-cultivation showed that the CCL-171 fibroblasts up-regulate the interferon response genes 2.8-fold on average, whereas the MDA-MB-231 breast cancer cell line up-regulates them about 11-fold. **(b) **Immunohistochemistry for STAT1. STAT1 expression in a normal breast (left panel) and in a breast cancer specimen (right panel). STAT1 is predominantly expressed in the malignant epithelial cells at the stromal interface in a centrifugal gradient.

We investigated in which cell the interferon-response genes were induced in response to heterotypic interaction by differentially labeling the epithelial cells and the fibroblasts with distinct fluorescent dyes prior to co-culture, then sorting them after co-culture using FACS. Comparing gene expression patterns of cells in monoculture with those of the same cell type after co-cultivation showed that in the CCL-171 fibroblasts, the interferon-response genes were induced on average by a factor of only 2.7 whereas in the MDA-MB231 breast cancer cell line these genes were induced 11-fold (Figure 3a). This result of a predominant induction in the tumor cell is in line with immunohistochemical evidence that *in vivo *the interferon- response genes *STAT1, EPSTI1 *[17] and *EIF2AK2 *[32] are expressed in the malignant epithelial cells and to a much lesser extent in the stroma. To test whether a soluble factor is sufficient to induce the interferon response genes or whether direct cell-cell contact is needed for their induction, we let the cells interact in transwell co-cultures at low serum conditions. In this setting, neither the MDA-MB231 breast cancer cell line nor the CCL- 171 fibroblasts showed induction of interferon response genes, indicating that close cell-cell contact is necessary for interaction. If the induction of interferon-response genes depended on short-range epithelial-mesenchymal interactions, we would expect to find the expression of interferon-response genes mainly at the tumor-stromal interface. To test this hypothesis we stained normal breast and breast cancer sections using antibodies specific for STAT1, the key transcriptional activator of the interferon-response genes, and itself a protein over-expressed in response to interferon stimulation (Figure 3b). No staining was evident in normal breast samples. In tumor tissue sections consisting of a homogenous tumor island surrounded by stroma, we typically observed a distinctive pattern of STAT1 expression concentrated at the periphery of the tumor islands, near the tumor-stroma boundary, supporting the idea that the interferon-response genes are induced preferentially in the tumor cells in closest proximity to the stromal cells. The gradient in the response further suggests involvement of a soluble factor acting over a short range.

### Induction of interferon in co-culture

**Figure 4:**
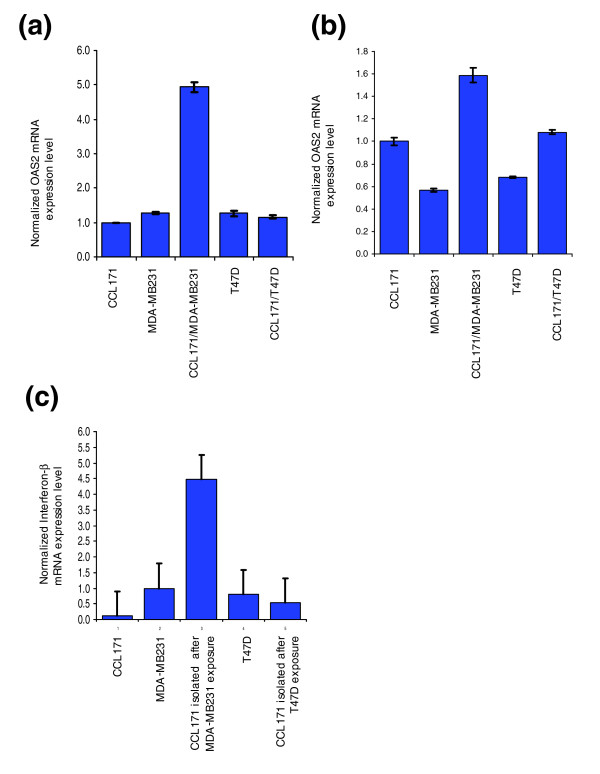
Induction of interferon response in two types of breast cancer cell lines. **(a) **MDA-MB231 cells were incubated in conditioned media from CCL-171 monoculture, MDA-MB231 monoculture, T47D monoculture, CCL-171/MDA-MB231 co-culture and CCL-171/T47D co-culture. *OAS2 *gene expression was determined by quantitative RT-PCR. The gene expression level of GAPDH was used for normalization between the samples. A strong induction of *OAS2 *by the supernatant from the CCL-171/MDA-MB231 co-culture can be seen in MDA-MB231. **(b) **Incubation of T47D cells with conditioned media from CCL-171 monoculture, MDA-MB231 monoculture, T47D monoculture, CCL-171/MDA-MB231 co-culture and CCL-171/T47D co-culture showed that only the CCL-171/MDA-MB231 co-culture supernatant induced *OAS2 *gene expression, although to a much lesser extent than in MDA-MB231 cells. **(c) **Gene expression levels of *IFNβ *were determined by quantitative RT-PCR. CCL-171 cells show much higher *IFNβ *expression levels when isolated by FACS after co-culture with MDA-MB231 than with T47D cells. Expression levels in tumor cells are shown as controls. The error bars show the standard deviation from the normalized mean.

To investigate the possible roles of soluble factors or direct cell-cell contact in triggering the observed interferon response, we tested the ability of conditioned medium from selected cultures to induce the response in a monoculture of MDA-MB231 cells. Conditioned medium from monocultures of either CCL-171 or MDA-MB231 cells did not induce interferon-response genes. However, conditioned media from an MDA-MB231/CCL-171 co-culture did induce the interferon response genes in MDA-MB231 cells. Thus, interferon-response genes are induced by a soluble factor, the induction depending upon direct contact between the tumor cells and fibroblasts. In contrast to the MDA-MB231/CCL-171 co-culture supernatant, the conditioned medium of the T47D/CCL-171 co-culture did not induce the interferon response genes when applied onto MDA-MB231 cells (Figure 4a). Conversely, when T47D cells were exposed to MDA-MB231/CCL-171 co-culture supernatant, the interferon-response was induced (Figure 4b). However, the response of the T47D cells to the co-culture supernatant was weaker than that of the MDA-MB231 cells. This implies that while the interferon-response genes can be induced in either tumor cell line, only the interaction of MDA-MB231 with fibroblasts released a soluble factor into the medium capable of inducing an interferon response. We speculated that the factor released by the fibroblasts might be a type I interferon. To confirm and localize the expression of type I interferon we used quantitative RT-PCR to analyze sorted cells after co-cultivation. We found over-expression of *IFNβ *in CCL-171 in response to interaction with MDA-MB231 but not in response to T47D (Figure 4c). Expression analysis of *IFNα *gave us the same result (data not shown), indicating that the expression of type I interferon genes by co-cultured fibroblasts might underlie the observed interferon response.

**Figure 5:**
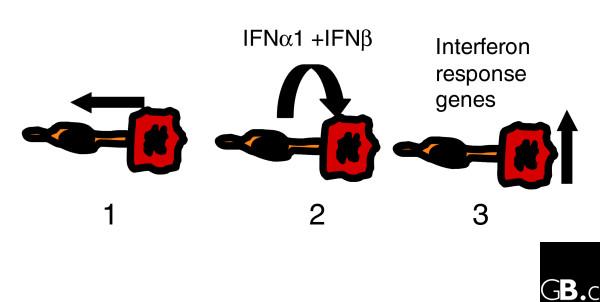
Model of interaction effects. Upon close cell-cell contact the tumor cells (red) interact with the fibroblasts (yellow) (1), which express type I interferon (*IFNα *and *IFNβ*) (2). They in turn induce the interferon response genes predominantly in the tumor cells (3).

Taken together, these results demonstrate that heterotypic interaction between fibroblasts and a specific subset of breast cancer cells can induce the fibroblasts to express type I interferons, resulting, in turn, in induction of interferon-response genes in the tumor cells and to a lesser extent in the fibroblasts (Figure 5). In our *in vitro *system, both estrogen-receptor positive and estrogen-receptor negative tumor cells are responsive to type I interferons, but the ability to induce expression of interferons in co-cultivated fibroblasts was specific to the estrogen-receptor negative breast cancer cells.

### Genomic analysis of epithelial-mesenchymal interaction effects in human cancers

**Figure 6:**
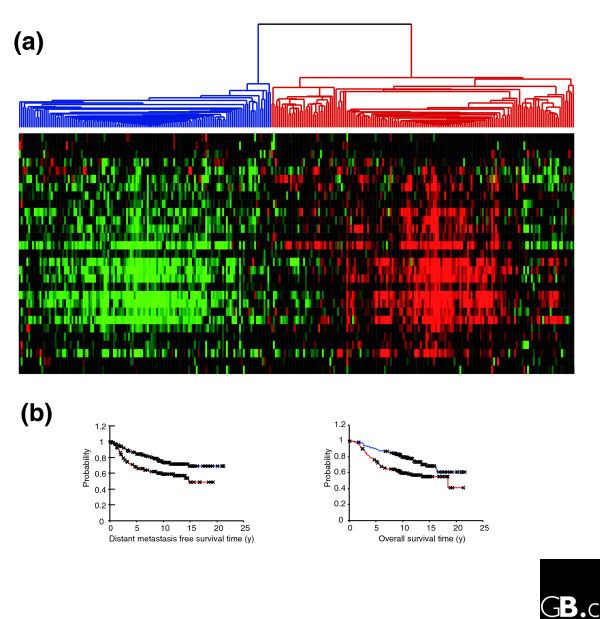
Interferon response gene expression in early stage breast cancer **(a) **The expression values of genes in the 'interferon response gene set' were extracted from a published expression study of 295 early stage breast cancers from the Netherlands Cancer Institute [35]. Genes and samples are organized by hierarchical clustering. The tumors segregated into two groups defined by high (red) or low (blue) expression levels of 29 genes matching the 'interferon response gene set'. **(b) **Correlation of interferon response with distant metastasis free and overall survival. Kaplan-Meier curves for the clinical outcomes of indicated tumors exhibiting high (red curve) and low (blue curve) interferon responses are shown.

**Figure 7:**
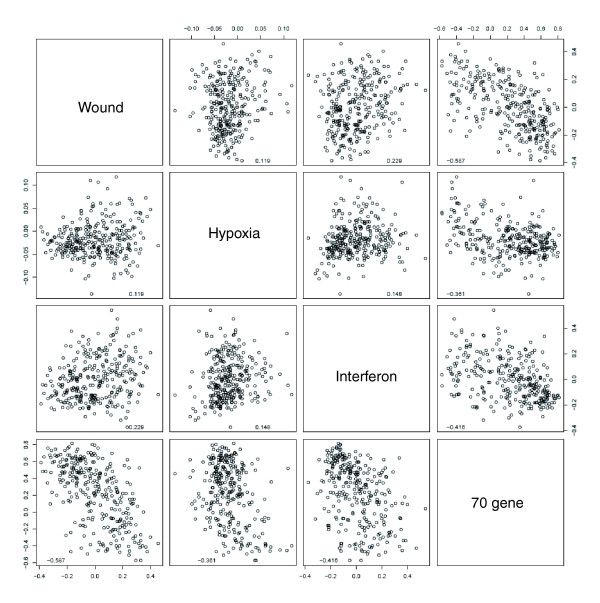
Correlation of the 70 genes signature [38], the wound signature [24], the hypoxia signature [25] and the interferon response score in the NKI dataset. Pairwise scatterplot-matrix of four gene signatures. Pearson correlations are shown in the lower part of each plot.

Interactions between cancer cells and non-malignant cells in the surrounding microenvironment are important determinants of cancer development and progression [11,14,33,34]. We reasoned that identifying and characterizing gene expression programs characteristically induced by interaction between specific pairs of cells in culture might enable us to recognize and interpret specific features in the expression profiles of human cancer that reflect similar interactions between tumor and stromal cells *in vivo*. The most consistent response to *ex vivo *co-cultivation of breast cancer and stromal cells was the induction of the interferon-response genes. We therefore looked for this response in the expression patterns in published data from 295 early stage (stage I and II) breast cancer samples from the Netherlands Cancer Institute (NKI) [35] (Figures 6a,b and 7). The interferon-response genes showed a strikingly coherent variation in expression among these cancers, enabling these cancers to be divided into two groups, one with relatively high expression and the other with relatively low expression of the interferon-response genes. Clustering the breast carcinomas based only on expression of the interferon response genes directed them into two main clusters, one with high-level expression of most of the interferon genes and the other with lower expression of these genes (Figure 6a). The same coordinated behavior and segregation of tumors could be observed in a different set of advanced breast cancer samples [36,37], suggesting that variation in this interferon-response program is a general feature in breast cancer (Additional data file 3).

As a first assessment of its potential biological relevance, we compared distant metastasis-free survival and overall-specific survival between the two groups distinguished by the interferon-response genes (Figure 6b). We found that tumors with high expression levels of interferon-response genes had a significantly shorter metastasis-free survival (*p *= 0.0014; 58% at 10 years) and overall survival (*p *= 0.001; 59% at 10 years) than tumors with low expression levels (metastasis free survival, 74% at 10 years; overall survival, 80% at 10 years).

The same trend toward unfavorable outcome in patients with cancers showing high levels of interferon-response gene transcripts (*p *= 0.067) could be seen in an analysis of published data from advanced-stage breast cancers [36,37]. As a metric that can be compared to known prognostic parameters and applied to other prospectively collected samples, we defined an 'interferon-response score' by averaging the gene expression levels for the 42 genes of the interferon-response gene list. The interferon response did not significantly correlate with clinical parameters such as age of the patient, tumor size, nodal stage or angio-invasion. It was, however, very significantly correlated with tumor grade and estrogen receptor status (*p *< 10^-6^; Additional data file 4), paralleling our *in vitro *findings that cell lines representing estrogen-receptor negative tumors preferentially induce the interferon-response genes in co-culture.

We also investigated the relationship between the interferon-response gene signature and three previously identified gene-expression signatures, which were useful prognosticators in this dataset. The first signature is a set of 70 genes [38], which was identified in a supervised analysis of a subset of the NKI early stage breast cancer dataset [35], to predict freedom from metastasis at 5 years. The second signature was identified *in vitro *by exposing fibroblasts to serum to mimic a wound response, and has been shown to predict risk of progression [39]. The third signature, the response to hypoxia *in vitro *[25], is also associated with a poor prognosis. The interferon signature was only very weakly correlated with either the wound signature or the hypoxia signature, and moderately correlated with the 70-gene prognostic profile, whereas the wound signature and the 70-gene score were more strongly correlated to one another (Figure 7). Thus, the interferon response appears to be a distinct feature of breast cancer biology, identifying a subgroup of cancers with a higher propensity for progression.

### STAT1 protein expression in a second independent breast cancer dataset

**Figure 8:**
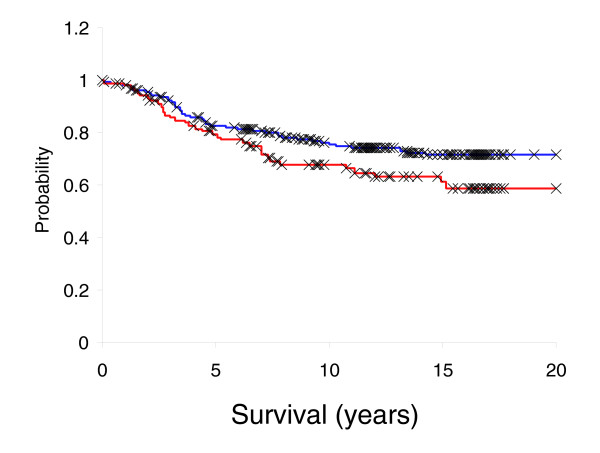
Immunohistochemical staining of STAT1 in a cohort of primary breast cancers: Kaplan-Meier disease-specific survival curve for 353 primary tumors assessed for STAT1. The red curve shows 102 patients bearing tumors with high STAT1 expression whereas the blue curve represents 251 patients with low or absent STAT1 expression. X = censored data.

As an independent test of the prognostic significance of interferon-response gene expression in primary early stage breast cancer we performed immunohistochemical staining for STAT1 in a tissue collection derived from a case series of women who underwent surgery for primary breast cancer at the Vancouver General Hospital between 1974 and 1995 [40]. Consistent with the variation we found in interferon-response gene expression in breast tumors, we found a large variability in the expression of STAT1 protein, the principal transcriptional regulator of the interferon response genes, in these tumors. Of the 353 primary tumors with interpretable results, 102 displayed high (28.9%), 184 low (52.2%) and 67 absent (18.9%) STAT1 expression. Paralleling the results from the NKI dataset, patients from Vancouver with tumors displaying high STAT1 expression levels had a higher risk of death due to breast cancer (33% dead from breast cancer at 10 years) than patients with tumors showing low or absent STAT1 expression (25% dead at 10 years) (*p *= 0.056; Figure 8).

## Discussion

The main objective of this study was to examine and characterize the effects of heterotypic cellular interaction, to gain insight into the underlying biology of these effects in normal mammary tissue and breast cancer. To isolate specific, direct interactions from more complex interactions involving multiple cell types in a whole tissue or organism we used a simple *ex vivo *co-culture model. Since some important heterotypic interactions can require direct cell-cell contact, we focused on a co-culture model where the two cell types were mixed. A challenge in the analysis of a mixed co-culture model is the separation of the interaction effects induced by signal exchange between the two cell types from the simple additive combination of their intrinsic gene expression patterns in the overall gene expression profile of the co-culture. Our strategy of normalizing for the simple additive effects based on a linear regression model proved to be advantageous, since it does not depend on prior knowledge of the exact proportional contribution of the different cell types to the superposed gene expression pattern. A similar approach has been described to define the proportional contribution of different cell cycle states in a mix of cells, although without taking into account interaction effects [41]. This strategy was effective in isolating the cell-cell interaction effects on gene expression.

We examined the effects on global gene expression of the molecular crosstalk between stromal fibroblasts and each of a diverse set of breast cancer cell lines or normal breast epithelial cells as they interact *in vitro*. Not unexpectedly, the picture of heterotypic interaction effects that emerged from combinatorial co-cultivation of multiple different cell types was complex, reflecting the different abilities of normal and malignant cells to send and to respond to extrinsic signals. The overall pattern of gene expression changes were dominated by the type of epithelial cells. Against our expectations, which were based on the knowledge that fibroblasts from different parts of the body show distinct gene expression patterns [42] leading to different physiological properties that persist through many passages of *in vitro *cultivation, the source of the fibroblast had only a minor influence on gene expression responses to heterotypic interaction in our co-culture system.

We cannot exclude the possibility that fibroblasts isolated from within a tumor might show additional specific interaction effects. Nevertheless, it would be surprising if carcinoma associated fibroblasts failed to show the strong effects that we consistently observed in co-cultures with fibroblasts of diverse origin. We recognize that these experiments might be insufficient to detect subtle differences between co-cultures involving different types of fibroblasts. To rigorously evaluate these differences a more extensive survey of co-culture conditions would be needed.

In our co-culture system a subset of tumor-stroma combinations showed induction of a set of genes characteristic of a 'myofibroblast' phenotype. In the same co-cultures, target genes of the TGF-β pathway were induced in parallel. This coordinated induction is in line with reports describing TGF-β as the major trigger of a 'myofibroblast' phenotype [29]. *In vivo*, activation of a contractile 'myofibroblast' phenotype in the tumor stroma occurs in a subgroup of patients, leading to shrinkage of the tumor environment causing skin dimpling and nipple retraction, both cardinal signs indicative of breast cancer. This example demonstrates how the analysis of heterotypic interaction effects allows inferring signaling pathways involved in specific physiological and morphological changes of importance in breast cancer.

The most prominent recurring theme arising from the heterotypic interactions we examined was the induction of an interferon-response program in cell lines derived from estrogen-receptor negative breast cancers upon co-culture with fibroblasts. Interferon-response genes showed a strikingly coordinated variation in expression in an analysis of diverse tumors and multiple datasets. Differential regulation of the interferon response genes has been observed in many human malignancies, including leukemias [43], ovarian cancer [44], gastric cancer [45], lung cancer [46] and breast cancer [30,43]. In breast cancer, in an attempt to validate the previously described intrinsic gene signatures [36,37], Hu *et al*. [47] assigned a small group of tumors with very high gene expression levels known to be induced by interferon as the 'interferon subtype' with a poor clinical outcome. Despite its common occurrence, the origin and the consequences of this phenomenon are unknown. Some reports have proposed that this program might reflect a viral infection or invasion of inflammatory cells in response to the tumor [43]. Our data suggest that the interferon response is not necessarily dependent on immune cells since our *in vitro *co-culture system comprises only fibroblasts and epithelial cells and no immune cells. Despite considerable effort to identify infective agents, we could not find any evidence for an infection in our cell culture causing the interferon response. Without excluding these possibilities, we propose that in a subset of breast cancer, the interferon response arises as an effect of the interaction of the malignant epithelial cells with the stroma.

At a first glance, the proposed link between interferon signaling and tumor-stroma interaction is surprising. However, interferons are pleiotropic cytokines, and while best known for their function as a viral defense mechanism they are also involved in other biological processes [48], such as the induction of cell cycle arrest, apoptosis, cell differentiation, immune stimulation and regulation of bone metabolism [49]. The induction of interferons at the interface between tumor cells and the surrounding stroma may have profound biological significance. In response to viral infection, induction of the interferon response genes, such as *EIF2AK2*, can lead to a global arrest of translation and subsequent apoptosis [50]. Interferon treatment has an anti-proliferative effect in some cultured cancer cells, and some human cancers shrink in response to interferon [51], leading to the speculation that an interferon response might be linked to a better prognosis [43]. In fact, our results show the opposite effect; patients with breast cancers displaying high interferon-response gene expression were 1.7 times more likely (95% confidence interval 1.1-2.6; *p *= 0.018) to develop metastasis and 1.8 times more likely to die of the disease (95% confidence interval 1.2-2.7; *p *= 0.006) than patients with tumors showing low expression levels of the interferon-response genes. Similar results have been reported by others. For example, an increase in EIF2AK2 expression and activity during tumor progression had been described in melanoma and colorectal cancer [52]. In breast cancer cells EIF2AK2 was elevated compared to normal breast epithelial cells [53]. Also, *IFI 27*, known to be inducible by IFNα, is frequently over-expressed in breast cancer [54]. IFITM1 over-expression in gastric cancer cells was reported to enhance migration and invasion *in vitro *[55]. These findings along with the observation that interferon response gene expression in cancer is highly coordinated, suggests the possibility that the interferon response program can promote cancer progression.

The role of STAT1, the main regulator of the interferon response genes, is controversial too. Our finding of a worse outcome for patients with tumors with high levels of STAT1 protein expression support our results of *STAT1 *mRNA expression levels and is in accordance with the expression levels of the full set of interferon response genes. The mechanism for the negative association between interferon response gene induction and patient outcome is not yet understood. Several mechanisms are possible. Up-regulation of STAT1 was found to be associated with resistance to radiotherapy [56]. IFITM1 was reported in another model to be involved in IFNα induced radioresistance [57]. For patients treated with radiotherapy, high expression of interferon response genes leading to radioresistance of the tumors could contribute to an unfavorable outcome compared to the more radiosensitive tumors. Since all patients receiving breast conserving therapy from the NKI dataset underwent adjuvant radiotherapy, this hypothesis cannot be further substantiated from our data because of the lack of an appropriate control group. Another possible mechanism, independent from an effect on therapeutic efficacy, could be mediated by an effect on invasiveness of the tumor. Up-regulation of STAT1 has been reported in breast cancer micrometastasis in the bone marrow [58], suggesting a more metastasis-prone phenotype.

The finding that an interferon response can be induced in response to tumor-stroma interaction raises questions for further inquiry. First, our results do not allow us to distinguish whether the interferon response has any role in contributing to tumor progression or is merely an incidental feature of certain cancers that tend to be more aggressive. If it should turn out that the interferon response has a contributing role in progression and metastasis in some tumors, therapeutic application of interferon might be detrimental in such cancers. Indeed, blocking the interferon response induction as a therapeutic target using antibodies or small molecule inhibitors might be beneficial in this situation. Second, apart from infectious agents, molecular signals that can induce interferon secretion are not well defined and the signals that induce interferon secretion in the stromal fibroblasts in our system are still to be discovered. One molecular mechanism for induction of interferon could be stimulation of a member of the toll like receptor family by a tumor cell associated ligand. Endogenous ligands for toll like receptors have been proposed, but further studies are needed to prove their existence [59]. In our experimental system close cell-cell contact was needed to induce the interferon-response, suggesting that a short-range signaling mechanism, perhaps involving a cell-surface ligand, might be involved.

Molecular interactions between epithelial and mesenchymal cells represent only a small part of the molecular conversation among all the interacting cells in the breast cancer microenvironment. The approach used in this work, employing an *ex vivo *model to develop gene expression signatures as an experimentally tractable window on the more complex interactions *in vivo *can be deliberately extended to other cells types, such as endothelial, inflammatory and immune cells. This technique may allow us to explore complex interactions among the multiple molecules operating in these cells to orchestrate the process of cancer progression and metastasis. Our experience suggests that *in vitro *modeling of specific processes and features of the tumor microenvironment can provide a valuable interpretive framework for analyzing the gene expression patterns in more complex heterogeneous *in vivo *samples and identify effects of heterotypic cellular interactions.

## Materials and methods

### Cell culture

HMECs (Cambrex Bio Science Walkersville, Inc.,Walkersville, MD, USA) were expanded in mammary epithelial basal medium supplemented with bovine pituitary extract, human epithelial growth factor, insulin and antibiotics (Clonetics, Cambrex Bio Science Walkersville, Inc.). MCF-7, T47D, MDA-MB231, MDA-MB436, SKBR-3, Hs578T, BT549, CCL-171, HTB-125 (ATTC) and HDF (Cambrex Bio Science Walkersville, Inc.) were propagated in DMEM supplemented with 10% FBS (HyClone, Logan, UT, USA), glutamine, 100 U/ml penicillin and 100 μg/ml streptomycin (GIBCO, Grand Island, NY, USA). For co-culture experiments the cells were cultivated for 48 h at 50,000 cells/cm^2 ^in endothelial basal media (Cambrex Bio Science Rockland, Inc., Rockland, ME, USA) with 0.2% FBS, which was a good universal medium for all cells involved. Separated co-cultures were kept in Transwell ^® ^chambers with a 0.4 μm pore size (Costar, Corning Inc., Corning, NY, USA). The cells for analysis were always harvested from the bottom well and reciprocal interactions were tested. Cells negatively tested for mycoplasma infection using MycoAlert™ (Cambrex Bio Science Rockland, Inc.) and VenorGem ^® ^(Sigma, Saint Louis, MO) mycoplasma detection kits used according to the manufacturers' instructions.

### Flowcytometry

Cells were fixed and stained using the Cytofix/Cytoperm™ Kit (BD Biosciences, San Jose, CA, USA) according to the manufacturer's instructions using 20 μg/ml STAT1a mAB (Abcam, Cambridge, MA, USA) and a fluorescein-5-isothyocyanate labeled goat anti-mouse IgG (Sigma-Aldrich, St Louis, MO, USA) for detection. Goat serum 1:200 was used for blocking. Analytical flow cytometry was done on a modified dual laser LSRScan (BD Immunocytometry Systems, San Diego, CA, USA) in the Shared FACS Facility, Center for Molecular and Genetic Medicine at Stanford, using FlowJo software (TreeStar, Ashland, OR, USA) for data analysis.

For FACS sorting, cells were stained with the lipophilic carbocyanine dye DiO (Vybrant ^® ^DiO cell-labeling solution, Molecular Probes™ Invitrogen, Eugene, OR, USA) in serum-free DMEM medium for 20 minutes and washed three times in calcium- and magnesium-free PBS according to the manufacturer's instructions before co-culturing. After 48 h, the cells were detached by incubation in Trypsin/EDTA (GIBCO, Grand Island, NY, USA) for 3 minutes and washed in ice-cold PBS and then immediately put on ice. Cell sorting was done on a MoFlow cell sorter (Becton Dickinson, Mountain View, CA, USA) in the Shared FACS Facility, Center for Molecular and Genetic Medicine at Stanford. The sorted cells were harvested in TRIZOL ^® ^LS Reagent (Invitrogen, Carlsbad, CA, USA). FlowJo software (TreeSTAR) was used for data analysis.

### RNA isolation and amplification

After discarding the culture medium and washing the cell layer once with PBS, total RNA was isolated by lysing the cells in the culture dish with RLT buffer (Qiagen, Valencia, CA, USA) and extraction with the RNeasy ^® ^Mini Kit (Qiagen). Total RNA (500 ng) was amplified using the Message Amp ™ II aRNA Kit (Ambion, Austin, TX, USA). The amplification product was checked for integrity by electrophoresis in a 1% agarose gel in MOPS buffer.

### cDNA microarrays and hybridization

We used human cDNA microarrays containing 40,700 elements that represent 24,472 unique genes based on Unique Clusters. Arrays were produced at the Stanford Functional Genomic Facility. Complete details regarding the clones on the arrays may be found at Stanford: functional genomics facility [60]. cDNA produced from 6 μg amplified RNA were hybridized to the array in a two-color comparative format, with the experimental samples labeled with one fluorophore (Cy5) and a reference pool of mRNA (Universal human reference, Stratagene, La Jolla, CA, USA) labeled with a second fluorophore (Cy3). Fluorescent dyes were purchased from Amersham Pharmacia Biotech (Piscataway, NJ, USA). Hybridizations were carried out using the standard protocol described previously [61].

#### Data analysis and clustering

Array images were scanned using an Axon Scanner 4000B (Axon Instruments, Union City, CA, USA), and image analysis was performed using Genepix Pro version 5.0 3.0.6.89 (Axon Instruments). The raw data files were stored in the Stanford Microarray Database [62]; the data used for the paper are available at the accompanying website [63]. Data were expressed as the log_2 _ratio of fluorescence intensities of the sample and the reference, for each element on the array.

The (Cy5/Cy3) ratio is defined in the Stanford Microarray Database as the normalized ratio of the background-corrected intensities. Spots with aberrant measurements due to obvious array artifacts or poor technical quality were manually flagged and removed from further analysis. A filter was applied to omit measurements where the fluorescent signal from the DNA spot was less than 50% above the measured background fluorescence surrounding the printed DNA spot in either the Cy3 or Cy5 channel. Genes that did not meet these criteria for at least 80% of the measurements across the experimental samples were excluded from further analysis. Valid data were filtered to exclude elements that did not have at least a three-fold deviation from the mean in at least three samples. Data were evaluated by unsupervised hierarchical clustering [64] and SAM [26] and displayed using Treeview [65].

### Determination of the heterotypic interaction effect on gene expression

To facilitate the identification of heterotypic interaction effects on global gene expression in a mixed co-culture experiment, we normalized the gene expression data based on the proportional contribution of each cell type to transcript abundance. Given that the average gene does not change due to heterotypic interaction and that there are simple additive effects to account for, we used a linear regression fit for normalization. To determine the contribution of each cell type to the combined gene expression pattern in the linear regression model, the expression levels of the monocultures are the predictors and the expression levels of the co-culture, the response.

Specifically, a set of equations (1-n) is established (one per gene), as illustrated in the additional data file 5 in which the expression level of gene n (e_n_, co-culture) in the mixed co-culture equals the fraction a of mRNA from cell type 1 times the relative expression level of gene n in type 1 mono-cultured cells plus the fraction (1-a) of mRNA from cell type 2 times the relative expression level of gene n in type 2 mono-cultured cells multiplied by the interaction coefficient I_n_. We assume that the average gene is not influenced by heterotypic interaction in the mixed co-culture represented as I = 1. Since the dataset over e_1-n _is skewed, we empirically identified a linear regression fit based on Gamma errors and identity link as a good model to calculate a. Then the equations 1-n can then be solved for I_1-n_, which results in a profile of interaction effects for the genes_1-n_. These interaction effects can be analyzed in much the same way as conventional gene expression measurements.

### Real time quantitative PCR

Total RNA (500 ng) was mixed with dT16 primer in a volume of 11 μl, incubated at 65°C for 10 minutes and immediately put on ice. Following addition of 100 units Superscript II reverse transcriptase (GIBCO, Carlsbad, CA, USA), reverse transcription was performed for 2 h at 42°C in 1× RT reaction buffer (GIBCO), 10 μM dithiothreitol, 500 μM dNTP (Amersham Biosciences, Pittsburgh, PA, USA) with 2.5 μM dT16 primer in a volume of 20 μl. PCR reactions were performed in a final volume of 20 μl with cDNA prepared from 20 ng RNA and a final concentration of 1× SYBR ^®^Green PCR Master Mix (ABI, Foster City, CA, USA) and 200 nM of each primer (sequences: GAPDH, forward GAAGGTGAAGGTCGGAGTC, reverse GAAGATGGTGATGGGATTTC; OAS2, forward GGAATACCTGAAGCCCTACGAA, reverse CCTGCAGACGTCACAGATGGT; IFNα, forward CCTCGCCCTTTGCTTTACTG, reverse GCCCAGAGAGCAGCTTGACT; IFNβ, forward ACCTCCGAAACTGAAGATCTCCTA, reverse TGCTGGTTGAAGAATGCTTGA). The reaction was run in an ABI 7700 Sequence Detection System with the following cycling conditions: 50°C for 2 minutes, 94°C for 10 minutes, then 40 cycles of 94°C for 15 s and 60°C for 60 s. For each gene a standard curve was prepared and triplicate measurements were performed for each sample.

### Immunohistochemistry

Large biopsy or tissue microarray sections were cut from paraffin blocks, deparaffinized in xylene, and hydrated in a graded series of alcohol. The slides were pretreated with citrate buffer and a microwave step. Immunostaining was performed using the DAKO Envision+ System, Peroxidase DAB, (DAKO, Cambridgeshire, United Kingdom) for STAT1a monoclonal antibody (1:100 dilution; Abcam).

We stained 1,024 tissue cores from 521 donor blocks. Immunohistochemistry images were acquired with the BLISS Microscope System (Bacus Laboratories, Lombard, IL, USA). Staining results were assessed using a three-point scoring system, where 0 = invasive tumor cells present in the tissue core and no staining seen, 1 = invasive tumor cells present with weak staining intensity and/or < 20% of the cells stained, and 2 = invasive tumor cells present with strong staining in > 20% of the cells. Tissue cores that failed to adhere to the glass slide, did not contain invasive carcinoma or were otherwise uninterpretable were excluded. Scoring of the arrays was analyzed using the Deconvoluter software as previously described [66], with each sample receiving the higher of the scores for two replicate cores.

### Human breast cancer dataset

The dataset for breast cancer contained 295 tumors analyzed on a 25,000 spot oligonucleotide array as described [35]. In brief, patients were diagnosed and treated at the NKI for early stage breast cancer (stage I and II) between 1984 and 1995. The clinical data were updated in January 2005. The median follow-up for patients still alive is now 12.3 years.

The interferon response gene list consists of 42 genes represented by 49 image clones on the cDNA Stanford array. Clones having the same Unigene locus were removed. The gene sequences were mapped to spots on the NKI array using Unigene build number 184 (released on 9 June 2005) to give 29 unique spots. In order to overcome possible overweighting of clones from Unigene clusters that were matched to more than one probe on the NKI array, expression values derived from probes that were not matched to the same Unigene cluster were averaged. Expression measurements for each gene were mean centered. The resulting dataset was subjected to hierarchical clustering with average linkage clustering [64] and displayed with Treeview [65].

Distant metastasis was analyzed as first event only (distant metastasis-free probability). If a patient developed a local recurrence, axillary recurrence, contralateral breast cancer or a second primary cancer (except for non-melanoma skin cancer), she was censored at that time and subsequent distant metastases were not analyzed. This is based on the theoretical possibility that the locally recurrent or second primary cancers could be a source for distant metastases. An ipsilateral supra-clavicular recurrence was soon followed by a distant metastasis in all but one patient. An ipsilateral supra-clavicular recurrence was thus considered the first clinical evidence for metastatic disease for this analysis and patients were not censored at the time of ipsilateral supra-clavicular recurrence. Overall survival was analyzed based on death from any cause and patients were censored at last follow up. Kaplan-Meier survival curves were compared by the Cox/Mantel log/rank test using Winstat for Microsoft Excel (RFitch Software, Staufen, Germany). Multivariate analysis by the Cox proportional hazard method was performed using the software package SPSS R 11.5 (SPSS, Inc., Chicago, IL, USA).

A dataset of gene expression patterns from advanced breast cancers was described by Sorlie *et al*. [36,37]. Expression data from 19 image clones representing the interferon response gene list were included in this dataset. Genes and samples were organized by hierarchical clustering. Relapse-free and overall survival were calculated as described above.

The independent breast cancer tissue microarray validation series is as described [40]; immunohistochemical scores for STAT1 were related to breast cancer-specific survival by Kaplan-Meier analysis with log-rank test.

## Abbreviations

DMEM, Dulbecco's modified Eagle's medium; EIF2AK2, eukaryotic translation initiation factor 2-alpha kinase; FACS, fluorescent assisted cell sorting; FBS, fetal bovine serum; FDR, false discovery rate; HMEC, human mammary epithelial cell; IFIT, interferon-induced protein with tetratricopeptide repeats; IFN, interferon; OAS, 2',5'-oligoadenylate synthetase; NKI, Netherlands Cancer Institute; PBS, phosphate-buffered saline; SAM, significance analysis of microarray data; STAT, signal transducer and activator of transcription; TGF, transforming growth factor.

## Authors' contributions

MB and POB designed the research; MB and RP performed the research; MB, TH and TN contributed new reagents/analytical tools; MB, DSAN, TH and POB analyzed the data; and MB and POB wrote the paper. The authors declare no conflict of interest

## Additional data files

The following additional data are available with the online version of this paper. Additional data file 1 is a table listing the interferon response genes. Additional data file 2 shows the expression of OAS2 measured by RT-PCR in the co-culture CCL171/MDA-MB-231 and the expression of STAT1 measured by immunofluorescent staining and FACS analysis. Additional data file 3 shows the analysis of the interferon response signature in advanced human breast cancers. Additional data file 4 shows box and scatter plots illustrating the correlation of the interferon score to clinical parameters with known prognostic significance. Additional data file 5 is an illustration of the linear regression model used to normalize for additive effects in the mixed co-culture gene expression data.

## Supplementary Material

Additional data file 1 Interferon response genes. Click here for file

Additional data file 2 Expression of OAS2 measured by RT-PCR in the co-culture CCL171/MDA-MB-231 and the expression of STAT1 measured by immunofluoresecent staining and FACS analysis. Click here for file

Additional data file 3 Analysis of the interferon response signature in advanced human breast cancers. Click here for file

Additional data file 4 Box and scatter plots illustrating the correlation of the interferon score to clinical parameters with known prognostic significance. Click here for file

Additional data file 5 Linear regression model used to normalize for additive effects in the mixed co-culture gene expression data. Click here for file
