# Supplementary material for: Characterization of heterotypic interaction effects in vitro to deconvolute global gene expression profiles in cancer
Source: Genome Biol. 2007 Sep 14;8(9):R191. doi: 10.1186/gb-2007-8-9-r191 (PMC2375029; doi:10.1186/gb-2007-8-9-r191)
Supplement: Additional data file 2 — Expression of OAS2 measured by RT-PCR in the co-culture CCL171/MDA-MB-231 and the expression of STAT1 measured by immunofluoresecent staining and FACS analysis. [file gb-2007-8-9-r191-S2.pdf]

Additional File 2

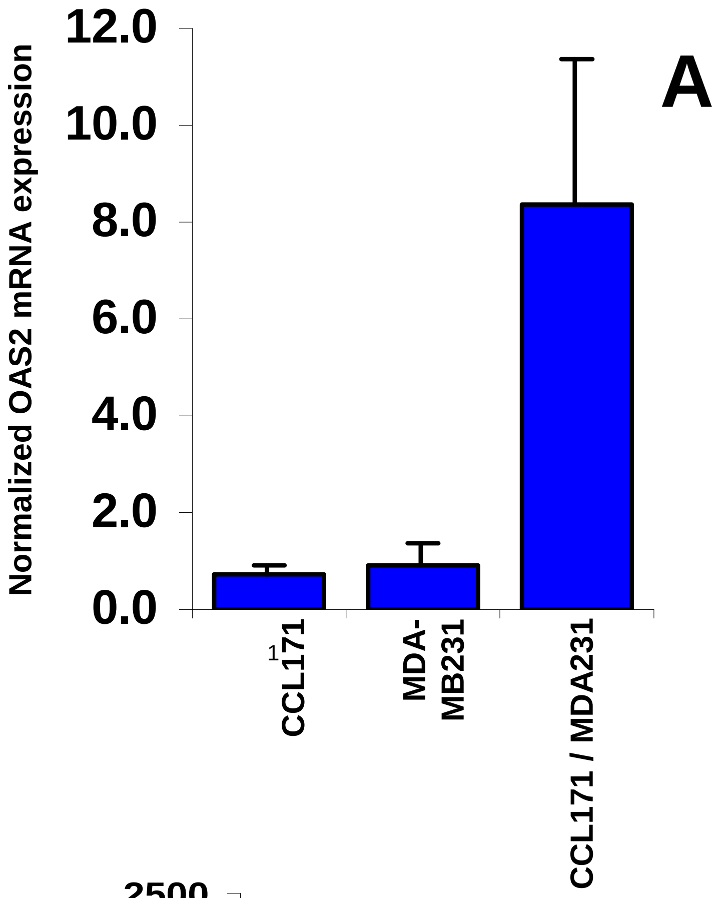

Legend Figure A:
Expression of OAS2 was determined by quantitative RT-PCR. Expression of GAPDH was used for normalization between the samples. OAS2 was up-regulated in CCL171/MDA-MB-231 co-culture compared to these cells cultivated as monocultures.

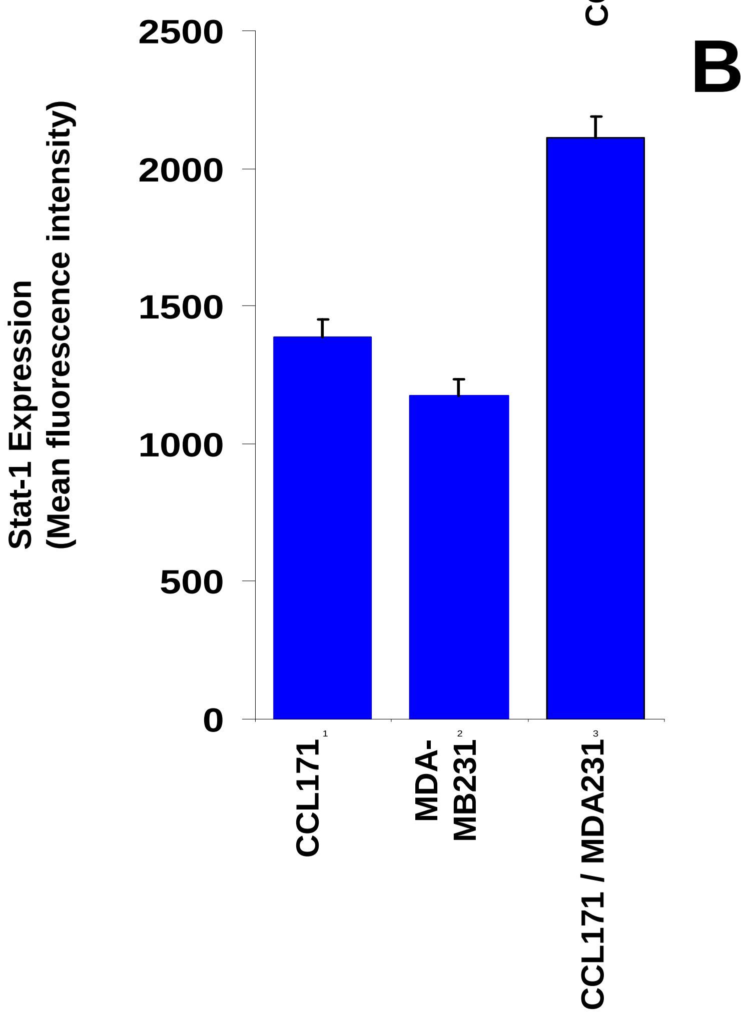

Legend Figure B:
Expression of STAT1 determined by immunofluorescent staining and FACS analysis.
