# Supplementary material for: Characterization of heterotypic interaction effects in vitro to deconvolute global gene expression profiles in cancer
Source: Genome Biol. 2007 Sep 14;8(9):R191. doi: 10.1186/gb-2007-8-9-r191 (PMC2375029; doi:10.1186/gb-2007-8-9-r191)
Supplement: Additional data file 3 — Analysis of the interferon response signature in advanced human breast cancers. [file gb-2007-8-9-r191-S3.pdf]

Additional File 3

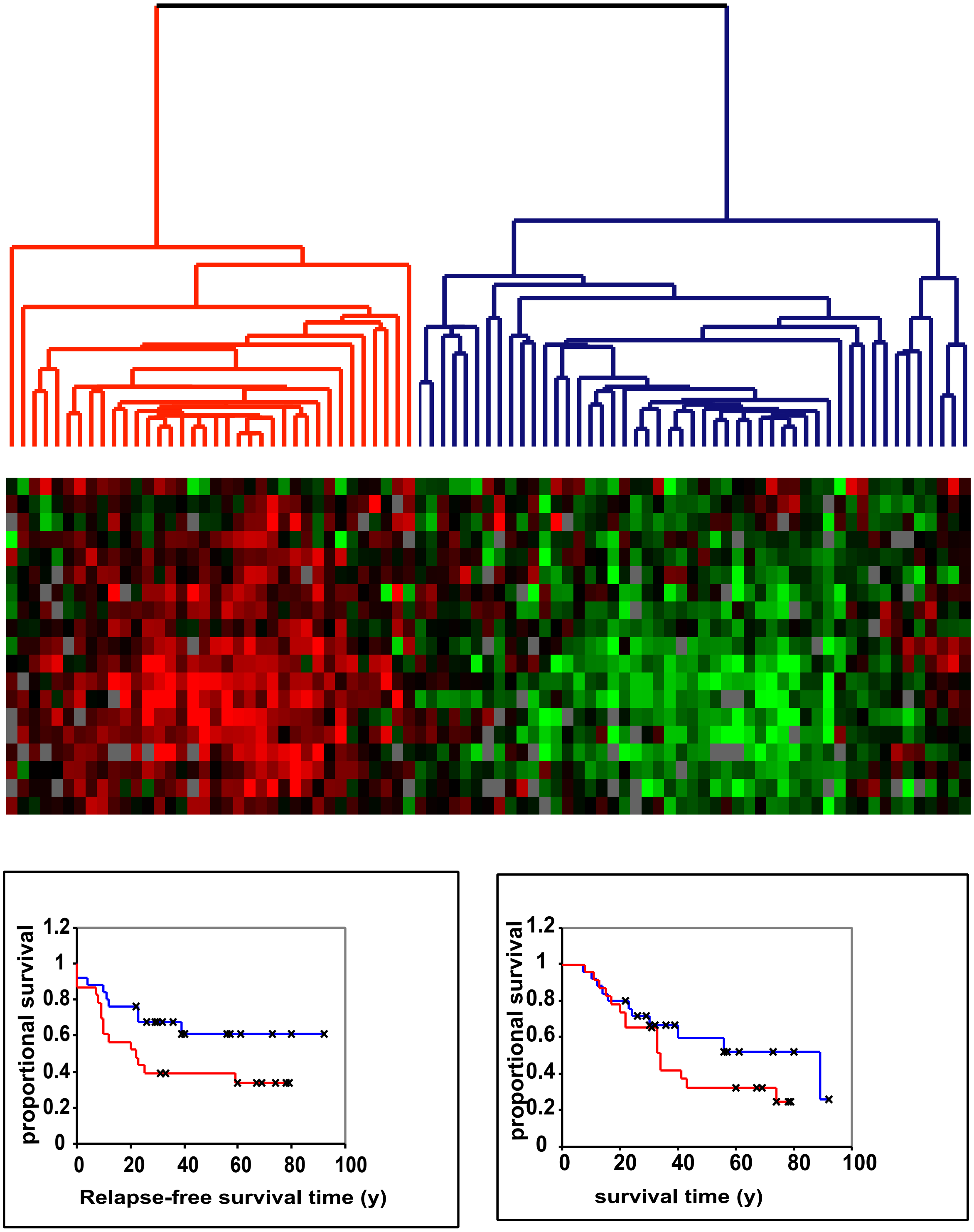

Legend:
The analysis of the interferon-response signature in advanced human breast cancers. Expression patterns of interferon-response genes in a group of breast carcinomas and normal breast tissue previously described in Sorlie et al.[36, 37]. Genes and samples were organized by hierarchical clustering. Kaplan-Meier survival curves for the two classes of tumors. Tumors with high transcript levels of the interferon response genes had worse relapse-free and overall survival compared to tumors with low interferon response gene expression.
