# Supplementary material for: Characterization of heterotypic interaction effects in vitro to deconvolute global gene expression profiles in cancer
Source: Genome Biol. 2007 Sep 14;8(9):R191. doi: 10.1186/gb-2007-8-9-r191 (PMC2375029; doi:10.1186/gb-2007-8-9-r191)
Supplement: Additional data file 4 — Box and scatter plots illustrating the correlation of the interferon score to clinical parameters with known prognostic significance. [file gb-2007-8-9-r191-S4.pdf]

Additional file 4

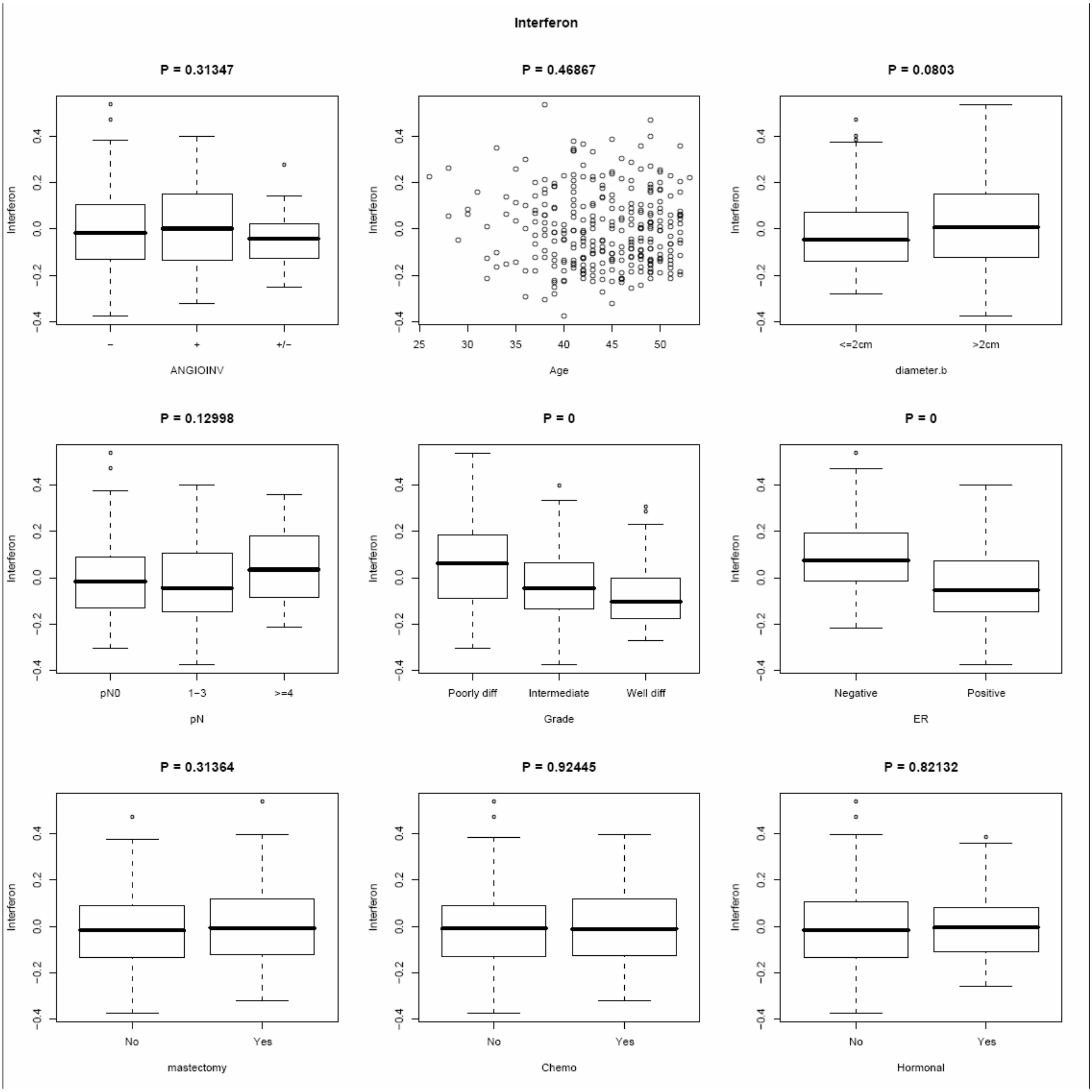

Legend:
Box and scatter plots showing the correlation of the interferon score to clinical parameters with known prognostic significance such as age, tumor diameter, nodal stage, histological grade, estrogen receptor expression, angio-invasion and therapeutic interventions such as type of surgery, chemotherapy and hormonal therapy.
