# Supplementary material for: Characterization of heterotypic interaction effects in vitro to deconvolute global gene expression profiles in cancer
Source: Genome Biol. 2007 Sep 14;8(9):R191. doi: 10.1186/gb-2007-8-9-r191 (PMC2375029; doi:10.1186/gb-2007-8-9-r191)
Supplement: Additional data file 5 — Linear regression model used to normalize for additive effects in the mixed co-culture gene expression data. [file gb-2007-8-9-r191-S5.pdf]

Additional file 5

Determination of interaction effect on gene expression

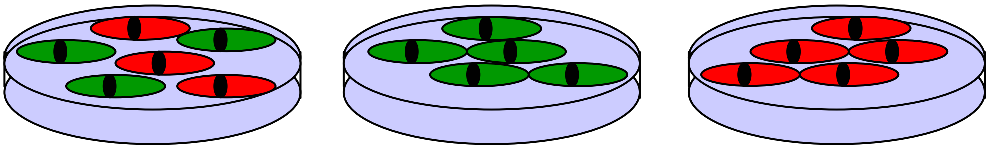

$$e_{1.co\text{-}culture} = \{(a \times e_{1.mono1}) + ((1\text{-}a) \times e_{1.mono2})\} \times I_1.$$
$$e_{2.co\text{-}culture} = \{(a \times e_{2.mono1}) + ((1\text{-}a) \times e_{2.mono2})\} \times I_2$$
$$e_{3.co\text{-}culture} = \{(a \times e_{3.mono1}) + ((1\text{-}a) \times e_{3.mono2})\} \times I_3$$
$$\vdots \qquad \vdots \qquad \vdots \qquad \vdots$$
$$e_{n.co\text{-}culture} = \{(a \times e_{n.mono1}) + ((1\text{-}a) \times e_{n.mono2})\} \times I_n$$

e = gene expression level (red/green ratio)

I = interaction effect on gene expression

a = fraction of mRNA from cell type 1

1. Average gene does not change: I=1
2. Solve for a and b using linear regression fit over e_1-e_n

3.. $$I_{1\text{-}n} = \frac{e_{1\text{-}n.co\text{-}culture}}{(a \times e_{1\text{-}n.mono1}) + ((1\text{-} a) \times e_{1\text{-}n.mono2})}$$

Legend:
Illustration of the linear regression model used to normalize for additive effects in the mixed co-culture gene expression data.
